# The management of type 2 diabetes before, during and after Covid-19 infection: what is the evidence?

**DOI:** 10.1186/s12933-021-01389-1

**Published:** 2021-10-01

**Authors:** Leszek Czupryniak, Dror Dicker, Roger Lehmann, Martin Prázný, Guntram Schernthaner

**Affiliations:** 1 Department of Diabetology and Internal Medicine, Medical University of Warsaw, Warsaw, Poland; 2 Department of Internal Medicine D, Hasharon Hospital, Rabin Medical Centre, Petah Tikva, Israel; 3 Sackler School of Medicine, Tel Aviv University, Tel Aviv, Israel; 4 Department of Endocrinology, Diabetes and Nutrition, University Hospital Zürich, Zürich, Switzerland; 5 3rd Department of Internal Medicine, 1st Faculty of Medicine, Charles University and General Faculty Hospital, Prague, Czech Republic; 6 Department of Medicine I, Rudolfstiftung Hospital Vienna, 1030 Vienna, Austria; 7 Medical University of Vienna, Vienna, Austria

**Keywords:** Covid-19, Type 2 diabetes, Glucose-lowering therapy

## Abstract

Patients with Covid-19 place new challenges on the management of type 2 diabetes, including the questions of whether glucose-lowering therapy should be adjusted during infection and how to manage a return to normal care after resolution of Covid-19 symptoms. Due to the sudden onset of the pandemic, physicians have by necessity made such important clinical decisions in the absence of robust evidence or consistent guidelines. The risk to patients is compounded by the prevalence of cardiovascular disease in this population, which alongside diabetes is a major risk factor for severe disease and mortality in Covid-19. We convened as experts from the Central and Eastern European region to consider what advice we can provide in the setting of type 2 diabetes and Covid-19, considering the evidence before, during and after infection. We review recommendations that have been published to date, and consider the best available—but currently limited—evidence from large observational studies and the DARE-19 randomized control trial. Notably, we find a lack of guidance on restarting patients on optimal antidiabetic therapy after recovering from Covid-19, and suggest that this may provide an opportunity to optimize treatment and counter clinical inertia that predates the pandemic. Furthermore, we emphasize that optimization applies not only to glycaemic control, but other factors such as cardiorenal protection. While we look forward to the emergence of new evidence that we hope will address these gaps, in the interim we provide a perspective, based on our collective clinical experience, on how best to manage glucose-lowering therapy as patients with Covid-19 recover from their disease and return to normal care.

## Introduction

Physicians are adapting to a new era in healthcare brought about by the Covid-19 pandemic.[1] Among the myriad of challenges faced is the management of patients for whom Covid-19 risk is elevated, especially given the epidemiological evidence that diabetes and cardiovascular disease are major risk factors for severe Covid-19 and mortality.[2]–[13].

As experts in diabetology from the Central and Eastern European region, we convened to consider what advice we can provide for clinicians managing patients with type 2 diabetes, including the large proportion of patients who also present with cardiovascular disease or high cardiovascular risk. We note that several recommendations are already published, albeit with some contradictions;[14]–[21] however, we do not feel there are sufficient data available for us to adjudicate between these recommendations or to make our own evidence-based recommendations. Furthermore, we are concerned about the lack of guidance on restarting patients on optimal antidiabetic therapy post-Covid-19, and the potential impact of clinical inertia with regards to optimizing glucose-lowering therapy.[22].

As the pandemic progresses, we expect more evidence to emerge that may help to inform diabetes management in a Covid-19 setting. A first step comes from a real-world report on Covid-19 mortality among patients with type 2 diabetes in the UK. This nationwide study suggested that the pandemic does not provide grounds to change overall glucose-lowering drug preferences.[23].

In this article, we summarize what is and what is not known about managing type 2 diabetes during the Covid-19 pandemic, discuss recommendations made to date, review the implications of the latest real-world evidence, and make our own suggestions for patients who are returning to normal care after recovery from Covid-19.

### Why does diabetes care need updating for the Covid-19 era?

In patients with diabetes, the rates of severe disease and mortality during Covid-19 infection are elevated compared with non-diabetic individuals.[2]–[12, 23] Several pathological mechanisms have been proposed to underlie this phenomenon, and have been detailed extensively elsewhere [24]–[31]. Hyperglycemia is also associated with mortality [32, 33], raising the possibility that long-term optimal glucose control in the general diabetes population may be of particular importance for reducing mortality risk for those patients who later contract Covid-19.

Epidemiological studies have identified factors other than hyperglycaemia that heighten risk among patients with diabetes who are infected with Covid-19; unsurprisingly, these include advanced age and systemic inflammation as well as comorbidities such as obesity, hypertension, coronary artery disease, chronic kidney disease, and a history of stroke or heart failure [1, 6, 10, 33]–[49]. The widespread prevalence of these comorbidities, and proposed pathological overlap between their pro-inflammatory and pro-coagulative states and Covid-19, has added new complexity to diabetes care [10, 24, 25, 35, 40, 50]–[56], as has the observation that in some patients inflammation may be ongoing after the resolution of acute Covid-19 [57]. Similarly, even after discharge, the risk of mortality or new onset major adverse cardiovascular events (MACE) may remain high [10]. Elderly patients with type 2 diabetes may be a particular concern, owing to the position of advanced age as the most significant risk factor for poor outcomes with Covid-19.

The challenge of managing diabetes [1, 58] and its comorbidities [59] while under pandemic (and, at times, lockdown) conditions adds to the burden placed upon physicians. Of particular concern, there may be more restricted access to new initiation of glucose-lowering drugs; [60] meanwhile, patients’ control of diabetes may worsen [61]. Covid-19 itself has been linked to fluctuating and elevated glucose levels that can be indicative of poor prognosis, and can be difficult to manage upon hospitalization [33, 47, 48, 62]–[65].

Inadequate glycaemic control can be a risk factor for the development of diabetic ketoacidosis (DKA), [66]–[68] which is typically seen in patients who are volume-depleted as a result of fever and inadequate fluid intake, [66] symptoms that can occur in Covid-19. By contrast, little is known about hypoglycaemia in patients with Covid-19; in non-Covid-19 settings, hypoglycaemia can be a significant risk factor for poor outcomes in intensive care [69].

In the case of the glucagon-like peptide-1 receptor agonist (GLP-1 RA) and sodium–glucose transporter 2 inhibitor (SGLT2i) classes, patients may miss out on the important cardiorenal benefits that these agents offer [22, 60]—attributes that may even warrant exploration for the treatment mild or moderate Covid-19. Access to cardiology services themselves may also be impeded. In the first wave of the pandemic, several studies reported reductions in acute cardiovascular hospitalizations that suggested delayed care, such as primary percutaneous coronary intervention (PCI) procedures in patients with diabetes and ST-segment elevation myocardial infarction (STEMI) [70]–[73]. Even after admission, testing for Covid-19 status can delay necessary treatment [74]. An international study also found a stark reduction in diagnostic procedures [75]. Collectively, estimates have suggested that the impact on cardiology services will lead to a large number of excess deaths as an indirect consequence of Covid-19.[76] It is hoped that access has subsequently improved, but some issues may still remain and could re-emerge in the event of new waves of large-scale hospitalizations.

Beyond diabetes and its immediate comorbidities, the overall physical and mental health of patients with diabetes can also decline due to the socioeconomic impacts of Covid-19, such as lockdown, economic hardship and bereavement; indeed, anxiety has increased in patients with diabetes [1]. Thus, some patients with type 2 diabetes are particularly vulnerable during the pandemic.

### What evidence and recommendations are available for glycaemic control in a Covid-19 setting?

**Fig. 1:**
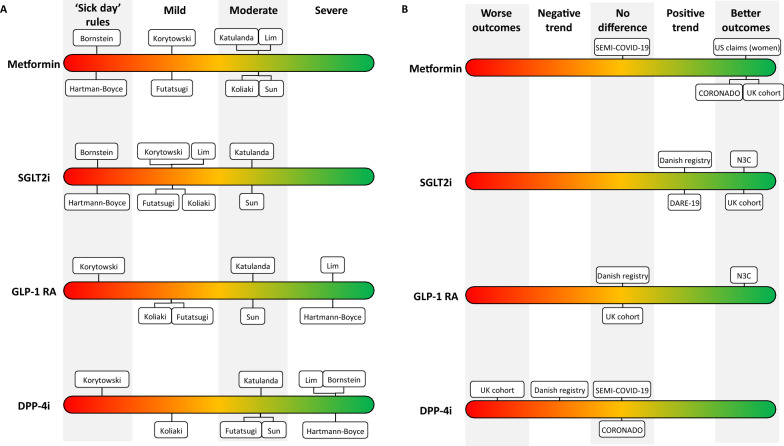
Recommendations and evidence for different classes of glucose-lowering drugs in patients with type 2 diabetes in a Covid-19 setting. **A** Recommendations for the management of type 2 diabetes in a Covid-19 setting have consistently advocated for the use of insulin (not shown) in patients hospitalized with severe Covid-19, but are less consistent in guidance for other glucose-lowering drug classes**.** Shown in this panel is the maximum severity of Covid-19 where various publications have recommended that metformin, SGLT2i, GLP-1 RA or DPP-4i can be continued in patients with type 2 diabetes. **B** Several large real-world studies have sought to compare mortality and other serious outcomes among glucose-lowering drugs in patients with Covid-19; however, these studies are limited by significantly different patient profiles between cohorts that may lead to confounding, even with the best efforts to control for biases. To avoid confounders, randomized controlled trials are need. One such study, DARE-19, has recently shown a trend towards positive outcomes with SGLT2i that was considered hypothesis generating, but was not statistically significant

Following a literature search, we have reviewed various recommendations [14]–[21] and reviews [77, 78] now available on managing diabetes in a Covid-19 setting (Table 1). While we applaud the efforts to rapidly disseminate advice in the fast-evolving circumstances of a novel virus pandemic, the challenge for physicians is that the guidance in some aspects lacks consistency (Fig. 1a) [14]–[21]. This is largely because of the many data gaps that mean we still cannot say definitively whether each glucose-lowering drug class has a negative, positive or neutral effect on Covid-19 outcomes. In particular, we find that there is minimal guidance on how to restart glucose-lowering therapies after temporary cessation due to Covid-19 [14]–[21, 77, 78].

**Table 1:** Summary of Covid-19 recommendations for glucose-lowering drugs in the management of type 2 diabetes

Publication	Metformin	SGLT2 inhibitors	GLP-1 RA	DPP-4 inhibitors	Insulin
Bornstein [21] April 2020	If patients are dehydrated, discontinue and follow sick day rules, due to risk of dehydration and lactic acidosis; monitor for chronic kidney disease or AKI	During illness, patients should stop taking the drugs and follow sick day rules, due to risk of dehydration and DKA; avoid initiating during respiratory illness; monitor for AKI	Dehydration should be closely monitored, and adequate fluid intake and regular meals encouraged	Can be continued, as generally well tolerated	Should be continued, with regular self-monitoring or CGM encouraged; early intravenous insulin therapy in severe courses (ARDS, hyperinflammation)
Katulanda [18] May 2020	Discontinue in severely ill patients with haemodynamic instability or hypoxia	Discontinue in patients where oral intake is not tolerated or who are severely ill	Discontinue in severely ill patients	May be continued in non-critically ill patients	Preferred treatment option in critically ill patients
Hartmann-Boyce [19] June 2020	Follow sick day rules and stop during acute illness	Follow sick day rules and stop during acute illness	Preferred treatment option in hospitalized patients, together with DPP-4 inhibitors and insulin	Preferred treatment option in hospitalized patients, together with insulin and GLP-1 RA	Preferred treatment option in hospitalized patients, together with DPP-4 inhibitors and GLP-1 RA
Korytowski [16] June 2020	Discontinue in hospitalized patients, due to the possibility of sudden and rapid deterioration in clinical status	Discontinue in hospitalized patients	Discontinue in patients hospitalized with acute disease	Generally not recommended in patients with acute disease, due to potential for abrupt deteriorations in clinical status; avoid saxagliptin and alogliptin due to higher risk for HF	Preferred treatment option in hospitalized patients
Koliaki [17] July 2020	Discontinue in patients who are hospitalized with severe disease and who have hypoxia and haemodynamic instability, due to the risk of lactic acidosis; monitor renal function	Continue in non-hospitalized patients with mild disease, due to significant cardiorenal protective effects; discontinue in patients who are hospitalized with severe disease, due to the risk of euglycemic DKA in the event of dehydration and insulinopaenia	Continue with caution in non-hospitalized patients with mild disease; discontinue in hospitalized patients with severe disease; consider dehydration risk due to GI adverse events; ensure adequate fluid and food intake	Continue in non-hospitalized patients with mild disease, due to favourable safety profile and suitability for a wide range of renal function	Should be continued; monitor serum potassium levels to prevent hypokalaemia
Futatsugi [20] October 2020	Continue in asymptomatic or mild Covid-19; should generally be discontinued in hospitalized patients	Consider discontinuing in patients at high risk of respiratory failure and thrombosis	As a precaution, discontinue in hospitalized patients due to possible GI adverse events that may worsen dehydration	Relatively safe to continue in mild-to-moderate Covid-19, but consider switching to insulin in severe disease	Preferred treatment option in hospitalized patients
Lim [15] November 2020	Not encouraged for use in critically ill patients, but can be used with caution; recommended in all other settings	Not recommended for moderate-to-severe (i.e. hospitalized) Covid-19, due to the potential for osmotic diuresis and possibly dehydration, which may be risk factors for AKI and DKA; can be used with caution in patients who are not hospitalized	Can be used with caution in critically ill patients; recommended in all other settings, especially due to cardiorenal benefits	Can be used in most patients, across a broad spectrum of Covid-19 severity, as generally well tolerated	Insulin is mainly recommended for critically ill patients with severe disease (as an infusion), but can be used in all patients
Sun [14] January 2021	Continue in mild-to-moderate Covid-19; avoid in critically ill patients	Continue in mild-to-moderate Covid-19; avoid in critically ill patients	Continue in mild-to-moderate Covid-19; more data needed to know whether suitable for acutely ill patients	Continue in mild-to-moderate Covid-19; more data needed to know whether suitable for acutely ill patients	Preferred treatment option for critically ill patients

There is also only limited, if any, guidance on how to de-intensify insulin regimens after discharge in patients initiated with insulin during hospitalization [14]–[21, 77, 78]—a scenario that has become more common due to the frequent need for insulin in patients hospitalized with Covid-19. For these patients, we can apply our existing knowledge from discharging patients in non-Covid-19 settings, as the principles are similar; the challenge is only to ensure that expertise on best practice is shared with the greater number of clinicians who may now be managing patients in such a scenario.

Overall, evidence is conflicting as to which glucose-lowering drugs are associated with the most favourable outcomes in patients with Covid-19 (Fig. 1b) and for the most part relies on observational data rather than randomized controlled trials. Large mortality studies have suggested either a benefit or no significant difference with metformin [23, 79]–[87] and SGLT2i; [39, 65]–[88] a benefit or neutral effect or even a negative effect with dipeptidyl peptidase-4 inhibitors (DPP-4i); [23, 89]–[101] a benefit or neutral effect with GLP-1 RA; [23, 88, 95] and a negative effect with insulin (Table 2) [23, 63, 102]–[104]. However, these observations may well be largely explained by confounders, and it is still not clear whether any of glucose-lowering agents provide true protection from Covid-19 [23, 88, 105]. Despite our reservations, the data do provide some reassurance on the lack of a safety signal, even for insulin where confounding factors were considered the likely explanation for the apparent negative effect [23].

**Table 2:** How notable studies reported the association of glucose-lowering drug classes with different outcomes in Covid-19

Study	Metformin	SGLT2 inhibitors	GLP-1 RA	DPP-4 inhibitors	Insulin
~ 3 million patients with type 2 diabetes, including ~ 13,500 who died due to Covid-19, in a UK nationwide population cohort [23]	Covid-19 mortality less likely vs no metformin	Covid-19 mortality less likely vs no SGLT2i	No significant difference in Covid-19 mortality vs no GLP-1 RA	Covid-19 mortality more likely vs no DPP-4i	Covid-19 mortality more likely vs no insulin
~ 12,500 patients with a positive Covid-19 test in a US longitudinal cohort (N3C) [88]	Not investigated	Lower rates of 60-day mortality, ER visits and hospitalization vs DPP-4i	Lower rates of 60-day mortality, ER visits and hospitalization vs DPP-4i	60-day mortality, ER visits and hospitalization more likely vs SGLT2i or GLP-1 RA	Not investigated
1970 people on GLP-1 RA, SGLT2i or DPP-4i in nationwide registries in Denmark who were infected with Covid-19 [95]	Not investigated	No significant differences to GLP-1 RA or DPP-4i, but a trend towards reduced 30-day mortality vs DPP-4i	No significant difference to SGLT2i	A non-significant trend towards increased 30-day mortality vs SGLT2i	Not investigated
2666 patients with type 2 diabetes in a nationwide Covid-19 registry in Spain (SEMI-COVID-19) [121]	No significant differences after propensity matching	Not investigated	Not investigated	No significant differences after propensity matching	No significant differences after propensity matching
2796 patients with diabetes hospitalized for Covid-19 in a nationwide study in France (CORONADO) [10, 82, 96]	28-day mortality less likely vs insulin or no metformin	Not investigated	Not investigated	Neutral effect vs patients not on DPP-4i [96]	28-day mortality more likely vs metformin
~ 25,000 patients on SGLT2i or DPP-4i in UK primary care [122]	Not investigated	Similar likelihood of Covid-19 onset vs DPP-4i	Not investigated	Similar likelihood of Covid-19 onset vs SGLT2i	Not investigated
Randomized control trial of dapagliflozin vs placebo in 1250 patients hospitalized with Covid-19 (DARE-19) [106]	Not investigated	No significant difference but trend towards reduced organ failure and death vs placebo	Not investigated	Not investigated	Not investigated
8121 patients hospitalized for Covid‐19 in a meta-analysis of five studies [123]	Mortality less likely vs no metformin	Not investigated	Not investigated	Not investigated	Not investigated
7008 patients with Covid‐19 and diabetes in a meta-analysis of nine studies [83]	Not investigated	Not investigated	Not investigated	In-hospital administration associated with reduced mortality vs no DPP-4i, but overall neutral effect	Not investigated
6256 patients with type 2 diabetes in a US claims database [79]	Mortality less likely vs no metformin (in women only)	Not investigated	Not investigated	Not investigated	Not investigated

Reported associations do not exclude confounding effects, and may be due to differences in patient characteristics rather than intrinsic drug properties

For SGLT2i, a recent randomized controlled trial provided further reassurance on safety. In the DARE-19 study, 1,250 patients hospitalized with Covid-19 at 95 sites in the US, Brazil, Mexico, Argentina, India, Canada and the UK, and who had cardiometabolic risk factors for developing serious complications, were randomized to receive dapagliflozin (an SGLT2i) or placebo between April 2020 and January 2021 [106]. Around half the patients had type 2 diabetes, and none had type 1 diabetes [106]. After 30 days, 11% of patients assigned to dapagliflozin and 14% of patients assigned to placebo had either died or suffered organ failure, most commonly respiratory or cardiovascular decompensation; looking at mortality only, the rates were 7% with dapagliflozin and 9% with placebo [106]. Although there was no statistically significant improvement in these outcomes, as had been hoped for, the investigators noted the trends favouring dapagliflozin, with numerically lower rates, and on that basis considered the results to be hypothesis generating [106].

As other endpoints were similar between the two groups, and overall adverse events were fewer with dapagliflozin than placebo, the DARE-19 study was at least able to demonstrate that the use of SGLT2i in patients hospitalized with Covid-19 is well tolerated and does not seem to elevate the risk for poor outcomes [106]. We would find it of interest to see further randomized controlled trials of SGLT2i, as well as GLP-1 RA, perhaps restricted to patients with mild-to-moderate Covid-19, with a view to preventing progression to severe disease or death.

Recommendations for managing glucose-lowering therapy have sought to discriminate between patients at risk of—but not infected with—Covid-19; patients who have mild disease that does not require hospitalization; patients who are hospitalized but not in critical care; and patients who are critically ill [14]–[21]. Based on these criteria, recommendations published to date have tended to agree on favouring insulin in hospitalized patients, where management of hyperglycaemia upon admission is a concern [14]–[21]. However, outside an intensive care setting, safe insulin dosing and glucose monitoring protocols are used inconsistently or not at all, which may result in increased risk of hypoglycaemia [107]. Safer treatments that could be considered include fixed-ratio formulations of GLP-1 RA or SGLT2i with basal insulin [107].

Some recommendations also note that DPP-4i can be considered in addition to insulin in the hospital setting, which may be highly relevant given the overlap in the elderly profile of DPP-4i recipients and patients hospitalized with Covid-19 [23].

Where recommendations disagree is on how to apply sick day rules for SGLT2i and metformin during Covid-19 infection, with some advocating for sick day rules with any degree of illness and others reserving this for hospitalized or even critically ill patients [14]–[21]. There is more agreement that no change to normal glucose-lowering treatment is needed in patients who may be at risk of infection but who are not currently infected.

If patients requiring hospitalization for Covid-19 continue on SGLT2i, they should be monitored for DKA, which can occur in patients with Covid-19 independently of treatment, but can sometimes also be associated with SGLT2i use [66]–[68, 108]–[116]. For patients with Covid-19 who are not hospitalized, we suggest that factors that may increase the risk for DKA are considered, namely fluid loss due to diarrhoea or low intake of food and drink due to a suppressed appetite or gastrointestinal symptoms. Patients on metformin with hypoxaemia or organ failure should be monitored for the risk of lactic acidosis, which can occur under these conditions [81].

Due to the paucity of data, all recommendations are based on clinical judgement alone, and made out of necessity without extensive, robust evidence. For this reason, and until such data are available, recommendations should be treated with caution and considered to be heavily caveated.

We are also mindful that the harm of suboptimal management of diabetes should not be overlooked, especially as inadequate glycaemic control has been linked to poor outcomes in patients with Covid-19 [52, 62]–[64]. Similarly, losing the cardiorenal protective effects and weight loss benefits of SGLT2i [117] and GLP-1 RA [22] may be detrimental given the cardiorenal risk posed by both type 2 diabetes and Covid-19, a question that remains open after the inconclusive results of the DARE-19 study [106]. Finally, while data on hypoglycaemia in patients with Covid-19 are limited, this should not deter clinicians from preferring therapies with a low risk of these events.

**Fig. 2:**
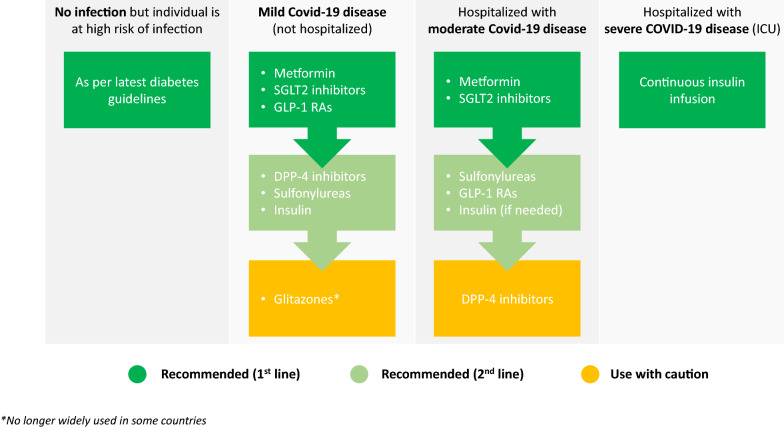
Suggested principles of glucose-lowering therapy for type 2 diabetes in a Covid-19 setting. In light of the limited available data, including observational studies and the DARE-19 trial, together with diabetes guidelines developed prior to the pandemic, we cautiously suggest these principles of glucose-lowering therapy in a Covid-19 setting. We emphasize that these suggestions are based on our clinical opinion and are not evidence-based, due to the paucity of evidence

Taking together our clinical experience and the review of literature discussed here, we suggest that the basic principles of glucose-lowering therapy for patients with type 2 diabetes in a Covid-19 setting could be directed as outlined in **Fig. ****2**.

### After Covid-19: how can we make sure that patients return to optimal control of type 2 diabetes?

Given that sick day rules and other recommendations may lead to treatment discontinuations in a Covid-19 setting, patients are at risk of losing the benefits of an optimized glucose-lowering drug regimen, especially if there is no systematic process for restarting treatment. However, for patients who had not previously been on a regimen optimized to the latest guidelines, treatment interruption or modification due to Covid-19 could also provide an opportunity to optimize glucose-lowering therapy upon recovery.

For those patients who do need to temporarily change their glucose-lowering regimen, the question remains as to when to de-intensify insulin and optimize treatment following recovery from Covid-19. More guidance is urgently needed to answer this question, but once again we are currently hampered by a lack of evidence.

Unfortunately, it is not straightforward to determine whether a patient has recovered from Covid-19; even once the infection has cleared, other sequalae may remain for some time, and risk of re-admission, MACE and even mortality remain high for a period; [118] it is not known whether this is a consideration for restarting glucose-lowering drugs. These difficulties are compounded by the overall strain placed on diabetes and cardiology services during the pandemic [1, 75] and the lack of guidance on post-Covid-19 care, with recommendations instead focusing on patients who are infected or at risk of infection [14]–[21]. The lack of guidance is of particular concern when bearing in mind that management of patients who are recovering from Covid-19 may be carried out, in most countries, by non-diabetes specialists.

Unless these challenges are overcome, patients may lose the benefit of their usual treatment regimen, optimized for well-controlled glycaemia and safety considerations; discontinuation may worsen glycaemic control and/or increase the likelihood of adverse effects such as hypoglycaemia. Furthermore, many patients with type 2 diabetes also have co-morbidities that would benefit from the various cardiorenal protective effects offered by agents within the SGLT2i and GLP-1 RA classes. Drug prescription data both prior to [22, 119, 120] and during [23] the Covid-19 pandemic has shown that many eligible patients do not receive SGLT2i or GLP-1 RA.

While any guidance should be received with caution, given the paucity of data, from our clinical experience and according to our literature review we would suggest that the basic principles of discharge could be directed as follows. Briefly, we advise that de-intensification should be considered at the time of discharge in patients with an intensified insulin regimen, taking into account efficacy, safety, patient burden, patient comfort and patient satisfaction with insulin treatment. The decision should be based on shared, informed decision-making with both the patient and other specialists, especially cardiologists and nephrologists, with whom specific comorbidities and special needs should be discussed, such as the benefits of medication offering cardiorenal protection, weight reduction or low risk of hypoglycaemia. The decision could thereby be perceived as an opportunity to improve patient outcomes beyond Covid-19, with optimized antihyperglycaemic treatment as per modern guidelines.

If it is decided that insulin should not be de-intensified at discharge, a timeline of 1 and/or 3 months should be set for re-assessment, which could involve telemedicine options such as online visits, telephone contact or remote access to data from glucose monitoring systems. Frequent monitoring and insulin down-titration are advised if the patient continues with a corticosteroid for Covid-19 following discharge from hospital.

## Conclusions

The sudden onset of the Covid-19 pandemic has forced diabetologists to make clinical decisions without the level of evidence that would normally be expected. As time progresses, some studies are beginning to shed light on the potential impact of various glucose-lowering drugs on outcomes such as mortality. However, these are mostly observational studies, while the DARE-19 trial had relatively modest patient numbers and inconclusive results. Notwithstanding these caveats, the emerging evidence suggests that no particular class of glucose-lowering drug carries an elevated risk; outside of an intensive setting, patients may be best served by continuing on their current regimens, with monitoring for DKA or lactic acidosis where appropriate.

While the danger posed by Covid-19 may be receding, the risks of poor glycaemic control and cardiorenal disease remain ever present [22]. Therefore, our hope is that management of type 2 diabetes during the pandemic will lead to new opportunities to optimize treatment to prolong life for patients and improve its quality.

## Data Availability

All data generated or analysed during this study are included in this published article.

## Abbreviations

AKI: Acute kidney injury
DKA: Diabetic ketoacidosis
DPP-4i: Dipeptidyl peptidase-4 inhibitor
GLP-1 RA: Glucagon-like peptide-1 receptor agonist
HF: Heart failure
MACE: Major adverse cardiovascular event
PCI: Percutaneous coronary intervention
SGLT2i: Sodium–glucose transporter-2 inhibitor
STEMI: ST-segment elevation myocardial infarction
UK: United Kingdom
